# Special Section: Metabolic Psychiatry

**DOI:** 10.1016/j.bpsgos.2023.08.017

**Published:** 2023-10-16

**Authors:** Deanna M. Barch

**Affiliations:** Departments of Psychological and Brain Sciences, Psychiatry, and Radiology, Washington University in St. Louis, St. Louis, Missouri

There is robust evidence about the critical interrelationships among nutrition, metabolic function (e.g., brain metabolism, insulin sensitivity, diabetic processes, and body weight, among other factors), inflammation, and mental health, a growing area of research now referred to as “metabolic psychiatry.” This special section of *Biological Psychiatry: Global Open Science* focuses on work outlining or contributing to our understanding of the associations among these metabolic factors and mental health, both in terms of risk factors for the development of mental health challenges and potential treatments for a range of psychiatric disorders, including work in both humans and animal models. The work represented in this special section spans the range from work in humans examining the potential role of metabolic factors in the onset or treatment of mental health conditions to preclinical models well suited to examine causal pathways between metabolic factors and behaviors and brain systems relevant for understanding the development and treatment of these disorders.

In a scoping review, Hiller *et al.* (1) provide an overview of previous research examining the utility of lipid disruptions as potential biomarkers in bipolar disorder, focusing on both variability in methods and key study findings. The current literature is mixed, with several study design issues that limit strong conclusions, including sample sizes, cross-sectional designs, and the presence of a range of potential confounds in studies, including physical activity and other health conditions. As such, while the potential for lipid markers to inform causes and treatments of bipolar disorder is still intriguing, this review suggests that improvements in study design and methods are needed to determine whether lipid-based markers have utility for understanding risk and treatment prediction in bipolar disorder.

In the context of major depressive disorder, Romankiewicz *et al.* (2) focused on the role of vascular diseases, such as hypertension and diabetes, in creating an increased risk for depression. This is a well-established relationship (3, 4, 5), but the causal mechanisms have not yet been identified. These authors tested the hypothesis that low-grade inflammation might be a potential mechanism, using prospective data from the UK Biobank to examine vascular risk factors (VRFs) and C-reactive protein associations to depression outcomes. VRFs at baseline predicted depression at follow-up, controlling for baseline depression, indicating that VRFs were harbingers of either new-onset or worsening depression. Baseline C-reactive protein did mediate this association, but the amount of variance accounted in the relation between VRFs and depression was relatively small. Thus, while these findings are consistent with the idea that inflammation-promoting effects of VRFs may contribute to depressive symptoms in midlife and late in life, it will be important to identify additional mechanisms contributing to the pathway between VRFs and depression. In related work, Sun *et al.* (6) examined the potential role of cholesterol metabolism in major depressive disorder. Specifically, they examined the expression of peripheral and brain-specific oxysterols and related gene polymorphisms in adults with major depressive disorder pre and post treatment and in healthy individuals. Individuals with depression expressed higher plasma levels of brain-secreted oxysterols. In addition, better treatment response over 12 weeks was associated with a reduction in both brain-secreted and peripherally secreted oxysterols. Individuals who did not respond to treatment had higher oxysterol levels than nonresponders post treatment. These findings raise the possibility of a potential role for markers of disrupted cholesterol metabolism as a marker of treatment response.

Much of the work on metabolic factors in mental health has focused on adults. However, there is a need to understand how cardiometabolic factors during pregnancy might be related to the brain and socioemotional development of offspring. Kwok *et al.* (7) addressed this question, finding that maternal markers of fasting glucose, low-density lipoprotein cholesterol, high-density lipoprotein cholesterol, and triglycerides were associated with increased conduct and hyperactivity problems over time in offspring. Intriguingly, there seemed to be stronger associations with maternal cardiometabolic risk in earlier pregnancy trimesters compared with later trimesters. This work is consistent with other evidence that maternal diets, such as high-fat diets, may be associated with increased risk of neurodevelopmental (8) and mental health outcomes in offspring (9). These data point to the need for more work on how maternal cardiometabolic health relates to child and adolescent development and the risk for mental health conditions.

In addition to metabolic factors potentially contributing to the development of mental health conditions, comorbid metabolic conditions may also present challenges to the effective treatment of psychopathology. For example, individuals who experience both depression and diabetes need additional treatments beyond conventional antidepressant treatment. Watson *et al.* (10) addressed this question using observational data and an approach that allowed them to emulate a randomized controlled trial. They specifically evaluated the relation of treatment with pioglitazone, an insulin-sensitizing drug, to antidepressant response among individuals with major depressive disorder and type 2 diabetes. They compared this form of adjunctive treatment to DPP4 inhibitors (non–insulin-sensitizing). Importantly, the combination of an antidepressant with pioglitazone was better than combination with DPP4 inhibitors in terms of antidepressant response as indexed by fewer treatment shifts over the next year, as well as fewer additions of antidepressants or antipsychotics. These findings suggest that targeted treatments are needed for individuals with major depressive disorder who have comorbid diabetes, providing evidence that adjunctive treatment with an insulin-sensitizing drug may be beneficial for such individuals.

In preclinical work addressing a related issue, Hühne-Landgraf *et al.* (11) focused on the ways in which circadian rhythms may impact both anxiety-like behaviors and metabolic function in mice, given that psychiatric and metabolic disorders often occur comorbidly in the same individuals. These authors examined what happened when they restored circadian rhythms in the suprachiasmatic nucleus, the circadian master pacemaker, of *Cry1/2-*deficient mice. These mice have disrupted circadian clocks in all tissues and display anxiety-like behavior and metabolic deficits. Intriguingly, the results indicated that virus-induced restoration of circadian rhythms in the suprachiasmatic nucleus reduced anxiety-like behaviors and improved glucose and energy metabolism in these mice. While in need of more investigation, these findings could suggest that restoration of disrupted circadian rhythms may be a potential therapy for treating comorbid psychiatric and metabolic disorders.

It has also been hypothesized that modulation of the gut microbiome might be an effective approach to the prevention or treatment of mental health conditions (12). This idea is based in part on the evidence that modulation of microbiome-gut-brain axis impacts emotional behavior (13). To provide data relevant to this key question, Schell *et al.* (14) examined the role of a probiotic, *Lactobacillus rhamnosus*, in a mouse model of diet-induced mood disorders. The authors found sex-specific effects, such that *Lactobacillus rhamnosus* impacted metabolism in females but anxio-depressive behavior in male mice. Interestingly, the reduction of depressive-like behavior by *Lactobacillus rhamnosus* in male mice was accompanied by changes in dopamine-related gene expression in the nucleus accumbens, suggesting altered dopamine signaling as a possible mechanism. These findings provide some evidence consistent with the idea that modification of the gut microbiome could have positive mental health impacts, but also indicate the importance of examining sex differences in relationships between the gut microbiome and mental health conditions (15).

Taken together, the articles in this special section provide new and intriguing data in both humans and animals that further support a link between metabolic factors and the development and treatment of mental health conditions across the lifespan. At the same time, these articles point to key gaps in the literature, including the need for studies with larger sample sizes with prospective designs that control for key confounds in human studies and more parallel human and animal studies that can be mutually informative in terms of causality and clinical relevance.

## References

[1] Hiller J.K., Jangmo A., Tesli M.S., Jaholkowsi P.P., Hoseth E.Z., Steen N.E., Haram M. (2023). Lipid biomarker research in bipolar disorder: A scoping review of trends, challenges, and future directions. Biol Psychiatry Glob Open Sci.

[2] Romankiewicz L., Schaare H.L., Nestler S., Villringer A., Blöchl M. (2023). Mediation of the association between vascular risk factors and depressive symptoms by C-reactive protein. Biol Psychiatry Glob Open Sci.

[3] Brostow D.P., Petrik M.L., Starosta A.J., Waldo S.W. (2017). Depression in patients with peripheral arterial disease: A systematic review. Eur J Cardiovasc Nurs.

[4] Mai A.S., Lim O.Z.H., Ho Y.J., Kong G., Lim G.E.H., Ng C.H. (2022). Prevalence, risk factors and intervention for depression and anxiety in pulmonary hypertension: A systematic review and meta-analysis. Front Med (Lausanne).

[5] Nouwen A., Adriaanse M.C., van Dam K., Iversen M.M., Viechtbauer W., Peyrot M. (2019). Longitudinal associations between depression and diabetes complications: A systematic review and meta-analysis. Diabet Med.

[6] Sun Z., Yang J., Zhou J., Zhou J., Feng L., Feng Y. (2023). Tissue-specific oxysterols as predictors of antidepressant (escitalopram) treatment response in patients with major depressive disorder. Biol Psychiatry Glob Open Sci.

[7] Kwok J., Khanolainen D.P., Speyer L.G., Murray A.L., Torppa M.P., Auyeung B. (2023). Examining maternal cardiometabolic markers in pregnancy on child emotional and behavior trajectories: Using growth curve models on a cohort study. Biol Psychiatry Glob Open Sci.

[8] Urbonaite G., Knyzeliene A., Bunn F.S., Smalskys A., Neniskyte U. (2022). The impact of maternal high-fat diet on offspring neurodevelopment. Front Neurosci.

[9] Monteiro S., Nejad Y.S., Aucoin M. (2022). Perinatal diet and offspring anxiety: A scoping review. Transl Neurosci.

[10] Watson K., Akil H., Rasgon N. (2023). Toward a precision treatment approach for metabolic depression: Integrating epidemiology, neuroscience, and psychiatry. Biol Psychiatry Glob Open Sci.

[11] Hühne-Landgraf A., Laurent K., Frisch M.K., Wehr M.C., Rossner M.J., Landgraf D. (2023). Rescue of comorbid behavioral and metabolic phenotypes of arrhythmic mice by restoring circadian *Cry1/2* expression in the suprachiasmatic nucleus. Biol Psychiatry Glob Open Sci.

[12] Freijy T.M., Cribb L., Oliver G., Metri N.J., Opie R.S., Jacka F.N. (2022). Effects of a high-prebiotic diet versus probiotic supplements versus synbiotics on adult mental health: The “Gut Feelings” randomised controlled trial. Front Neurosci.

[13] Ke S., Guimond A.-J., Tworoger S.S., Huang T., Chan A.T., Liu Y.-Y., Kubzansky L.D. (2023). Gut feelings: Associations of emotions and emotion regulation with the gut microbiome in women [published online Mar 21]. Psychol Med.

[14] Schell M., Wardelmann K., Hauffe R., Rath M., Chopra S., Kleinridders A. (2023). *Lactobacillus rhamnosus* sex-specifically attenuates depressive-like behavior and mitigates metabolic consequences in obesity. Biol Psychiatry Glob Open Sci.

[15] Holingue C., Budavari A.C., Rodriguez K.M., Zisman C.R., Windheim G., Fallin M.D. (2020). Sex differences in the gut-brain axis: Implications for mental health. Curr Psychiatry Rep.

